# Reintroducing Heart Sounds for Early Detection of Acute Myocardial Ischemia in a Porcine Model – Correlation of Acoustic Cardiography With Gold Standard of Pressure-Volume Analysis

**DOI:** 10.3389/fphys.2019.01090

**Published:** 2019-08-22

**Authors:** Marco Luciani, Matteo Saccocci, Shingo Kuwata, Nikola Cesarovic, Miriam Lipiski, Patricia Arand, Peter Bauer, Andrea Guidotti, Evelyn Regar, Paul Erne, Michel Zuber, Francesco Maisano

**Affiliations:** ^1^Department of Cardiovascular Surgery, University Hospital Zurich, Zurich, Switzerland; ^2^Division of Surgical Research, University Hospital Zurich, Zurich, Switzerland; ^3^VisCardia, Inc., Portland, OR, United States; ^4^Faculty of Biomedical Sciences, Università della Svizzera Italiana, Lugano, Switzerland; ^5^Department of Cardiology, University Hospital Zurich, Zurich, Switzerland

**Keywords:** acute coronary syndrome, acute myocardial ischemia, heart sound, hemodynamics, animal model

## Abstract

**Background:**

Acoustic cardiography is a hybrid technique that couples heart sounds recording with ECG providing insights into electrical-mechanical activity of the heart in an unsupervised, non-invasive and inexpensive manner. During myocardial ischemia hemodynamic abnormalities appear in the first minutes and we hypothesize a putative diagnostic role of acoustic cardiography for prompt detection of cardiac dysfunction for future patient management improvement.

**Methods and Results:**

Ten female Swiss large white pigs underwent permanent distal coronary occlusion as a model of acute myocardial ischemia. Acoustic cardiography analyses were performed prior, during and after coronary occlusion. Pressure-volume analysis was conducted in parallel as an invasive method of hemodynamic assessment for comparison. Similar systolic and diastolic intervals obtained with the two techniques were significantly correlated [Q to min dP/dt vs. Q to second heart sound (*r*^2^ = 0.9583, *p* < 0.0001), PV diastolic filling time vs. AC perfusion time (*r*^2^ = 0.9686, *p* < 0.0001)]. Indexes of systolic and diastolic impairment correlated with quantifiable features of heart sounds [Tau vs. fourth heart sound Display Value (*r*^2^ = 0.2721, *p* < 0.0001) cardiac output vs. third heart sound Display Value (*r*^2^ = 0.0791 *p* = 0.0023)]. Additionally, acoustic cardiography diastolic time (AUC 0.675, *p* = 0.008), perfusion time (AUC 0.649, *p* = 0.024) and third heart sound Display Value (AUC 0.654, *p* = 0.019) emerged as possible indicators of coronary occlusion. Finally, these three parameters, when joined with heart rate into a composite joint-index, represent the best model in our experience for ischemia detection (AUC 0.770, *p* < 0.001).

**Conclusion:**

In the rapidly evolving setting of acute myocardial ischemia, acoustic cardiography provided meaningful insights of mechanical dysfunction in a prompt and non-invasive manner. These findings should propel interest in resurrecting this technique for future translational studies as well as reconsidering its reintroduction in the clinical setting.

## Introduction

Cardiac auscultation has been a pivotal part of patients’ examination since the stethoscope invention in 1816 by the French physician René Laennec. Despite technical refinements, knowledge advancements and limited costs, auscultation has lost momentum over time in favor of less operator-dependent techniques. In fact, findings obtained from acoustic examination can be affected by operator’s expertise, limited sensitivity of the human ear and lack of reproducibility.

Nevertheless, by coupling heart sound recordings together with ECG tracings, investigators can acquire important insights regarding the electrical-mechanical activity of the heart in an unsupervised, non-invasive and inexpensive manner. Such a hybrid technique, acoustic cardiography (AC) (Erne, 2008), has been proven as a reliable method for patient stratification and prognosis in diverse pathological conditions [pulmonary arterial hypertension (Chan et al., 2013), atrial fibrillation (Dillier et al., 2010), coronary artery disease (Zuber and Erne, 2010; Thomas et al., 2017), acute myocardial ischemia (AMI) (Lee et al., 2009), premature ventricular contractions (Walia et al., 2019), heart failure with either preserved or reduced ejection fraction (Wang et al., 2013, 2016)].

In the specific setting of acute coronary syndrome, AC might play a role in patient management by detecting cardiac dysfunction (Marcus et al., 2005) in a prompt and accurate manner. For this reason we employed a porcine model of permanent acute coronary occlusion, and evaluated a possible correlation between parameters obtained by AC and the ones obtained with a reliable yet invasive technique, pressure-volume (PV) assessment. The present study aims to identify mechanical alterations of physiological significance such as indexes of contractile and relaxation function as well as systolic and diastolic time intervals (STIs and DTIs, respectively) occurring during AMI and detected with two different techniques (AC and PV measurements) in both stable and rapidly evolving conditions (prior and upon coronary occlusion) (Lewis et al., 1977).

## Materials and Methods

### Study Population

#### Ethical Committee

The animal housing and experimental protocols were approved by the Cantonal Veterinary Department, Zurich, Switzerland, under license no. ZH 219/2016, and were in accordance with Swiss Animal Protection Law. Housing and experimental procedures also conformed to the European Directive 2010/63/EU of the European Parliament and of the Council on the Protection of Animals used for scientific purposes and to the Guide for the Care and Use of Laboratory Animals (Institute of Laboratory Animal Resources, National Research Council, National Academy of Sciences, 2011).

#### Animal Care

Ten female Swiss large white pigs (50 ± 2 kg) were included in this study. All animals were pre-medicated with an intramuscular injection of 15 mg/kg BW Ketamine (Ketasol-100; Dr. E. Graeub AG, Berne Switzerland), 2 mg/kg BW Azaperone (Stresnil, Provet AG, Lyssach Switzerland) and 0.5 mg/kg BW Atropine sulfate 0.1%. All animals were intubated and ventilated with an inspired oxygen fraction (FiO_2_) of 0.5, tidal volume of 6–8 mL/kg, a frequency of 20 breaths per min, and a positive end-expiratory pressure (PEEP) of 5 cm H_2_O. Anesthesia was maintained with 0.6–1% isoflurane and deepened in accordance with the study protocol with a continuous infusion of propofol (2–5 mg/kg/h) (Propofol Lipuro 1%; Braun Medical AG, Sempach, Switzerland).

### Experimental Procedures

#### Porcine Model of Coronary Occlusion

**FIGURE 1:**
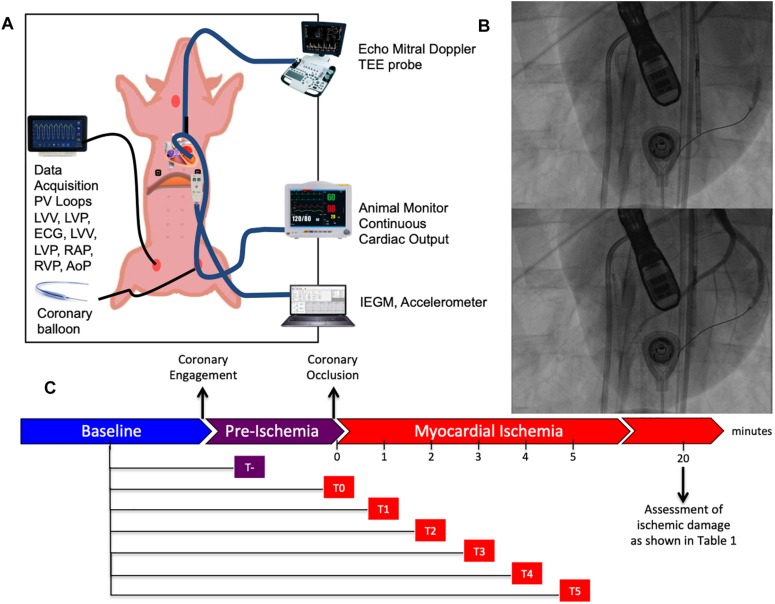
Experimental setup. **(A)** Schematic representation of the animal experiment. The swine is placed in supine position and instrumented with a series of different probes and catheters allowing multimodal realtime recordings. **(B)** Representative fluoroscopic images of myocardial infarction generation before (above) and after (below) balloon inflation. **(C)** Timeline of our study composed of three distinct phases: baseline, pre-ischemia (falling between coronary engagement and balloon inflation) and myocardial ischemia. Continuous recordings were performed through the pre-ischemia and myocardial ischemia for 5 min after coronary occlusion.

Continuous myocardial ischemia was induced by complete occlusion of the left circumflex artery distal to second marginal branch employing an adequately sized PCI balloon [Falcon CTO, PTCA Balloon, Invatec S.p.A. Roncadelle (BS), Italy]. Both AC and PV assessment were simultaneously conducted in supine position before, during and after coronary occlusion. Figure 1 provides an overview of our experimental setup and working model.

#### Cardiac Ultrasound Assessment

Echocardiography was performed with a Philips IE 33 machine (Philips Ultrasound, Andover, MA, United States) and its 3D-transesophageal probe. All data were stored digitally and evaluated by one person (MZ). After optimal placement of the transesophageal probe in a four chamber view, the probe was not moved throughout the study. Firstly, a 3D dataset for volume and ejection fraction analysis was registered. Secondly, transmitral flow was aquired with Pulsed Wave (PW) Doppler [Evmax (cm/s), Tdec time (ms), E-VTI (cm), Avmax (cm/s), A-VTI (cm)] and tissue velocities of medial mitral anulus with PW-Doppler [Eamax (cm/s), Ea-VTI (cm), Aamax (cm/s), Aa-VTI (cm), systolic vmax (cm/s), systolic-VTI (cm)] after optimization to a most perpendicular view as possible. E/A and E/E’ ratios were obtained by dividing Evmax by Avmax and Eamax, respectively.

#### Acoustic Cardiography Assessment

Acoustic cardiography was performed with AUDICOR (Inovise Medical, Inc., Portland, OR, United States) allowing simultaneous dual acquisition of heart sounds and ECG signals. The sensor was placed approximately between V2 and V4 lead location. Raw data were recorded and transferred to Inovise Medical Inc., and subsequently analyzed by the computerized algorithm for the measurement of ECG, heart sounds and systolic and diastolic interval times.

The following parameters were conceived and employed for our study. Intensities and persistence of heart sounds S1 (mitral valve closure), S2 (aortic valve closure), S3 (diastolic sound of limited ventricular longitudinal expansion), and S4 (diastolic sound of stiffness) were recorded and a derivative parameter called Display Value (DV) was generated. The AUDICOR automated heart sound algorithm is a hidden Markov model with inputs derived from a 6-level stationary wavelet transformation of the acoustic signal and from select fiducial times of the simultaneous ECG signal. The output includes a segmentation of the acoustic signal into individual heart sounds, as well as measurements derived from that segmentation. The S3 and S4 DV parameters are a combination of intensity and persistence of the acoustic signal within the segments appropriate in timing for those heart sounds. S3 and S4 DV is expressed on a 0 to 10 scale where if the DV is =5.0 a third or fourth heart sound is considered present. STIs were as follows: the Q-S1 interval is the time from the onset of the Q-wave on the ECG to the mitral valve closure (peak of the first heart sound or S1), also known as electromechanical activation time (EMAT). Left ventricular systolic time (LVST) is defined between (S1) and aortic valve closure (peak “aortic component” of the second heart sound or S2). Electromechanical systolic time (AC EMST) is defined by the time-window between the Q-wave onset and the aortic valve closure (S2). Concerning DTIs, the following parameters were employed: diastolic time (DT) as the time between aortic valve closure (S2) and the next mitral valve closure (S1), perfusion time (PT) as the interval between S2 and the next Q-wave onset.

#### Pressure-Volume Assessment

Simultaneous pressure-volume (PV) measurements were performed with a conductance catheter (CD Leycom, Zoetermeer, Netherlands). The conductance method calculates continuous volume tracings by measuring parallel electric conductance between adjacent electrodes. Correct positioning was verified by fluoroscopy and by inspecting segmental signals. Volume calibration was obtained by transoesophageal 3D echocardiography and/or by injection of NaCl hypertonic boluses. Data analysis was performed with dedicated data acquisition and analysis software (Conduct NT, CD Leycom). Along with real-time pressure, volumes, and a series of normally employed derivative parameters regarding myocardial contractility (ejection fraction, stroke work, cardiac output, minimal and maximal dP/dt, isovolumic relaxation constant Tau) additional intervals were conceived for our comparison. STIs were defined as follows: one interval starting from the onset of the Q wave until max dP/dt (Q to max dP/dt) also known as Pre-Ejection Period (PEP), and a longer one lasting until min dP/dt (Q to min dP/dt) (PV EMST). Additionally, the time window between maximal and minimal dP/dt values was also calculated. A DTI called diastolic filling time (DFT) was defined as the interval between dP/dt min and diastole’s end according to the Conduct NT manual.

### Data Analysis

#### Correlation Analysis

**FIGURE 2:**
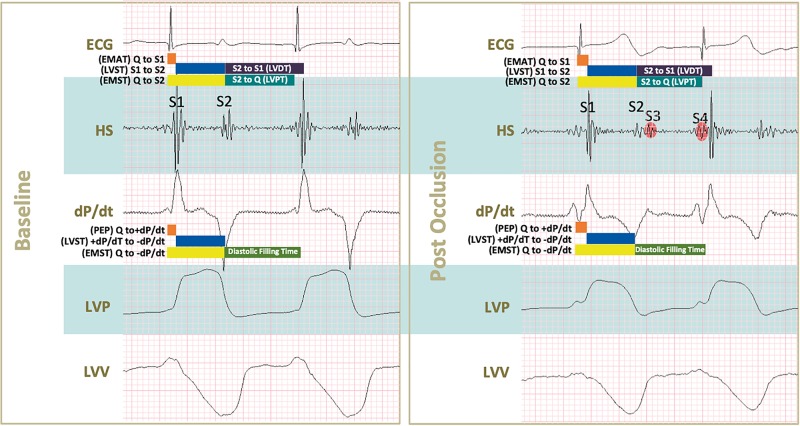
Conceived acoustic and pressure-volume derived parameters during baseline and after coronary occlusion. Acoustic systolic times were defined as follows: Q to S1 (electromechanical activation time or EMAT), S1 to S2 (left ventricular systolic time or LVST), and Q to S2 (electromechanical systolic time or EMST). Acoustic diastolic times were S2 to S1 (left ventricular diastolic time or LVDT), S2 to Q (left ventricular perfusion time or LVPT). Pressure-volume derived systolic times were: Q to +dP/dt (pre-ejection period), +dP/dt to –dP/dt (left ventricular systolic time) and Q to –dP/dt (electromechanical systolic time). The systolic time obtained by pressure-volume was delimited within –dP/dt to diastole’s end according to the company booklet. Upon occlusion a series of changes in shown tracings can be noticed: S1 and S2 intensities decrease, S3 and S4 start appearing, both maximal dP/dt and minimal dP/dt absolute values decrease indicating systolic and diastolic impairment, respectively. In addition, smaller excursions of left ventricular pressures and volumes (LVP and LVV) of tracings translate into lower systolic pressure generation and increased end-systolic volumes.

**FIGURE 3:**
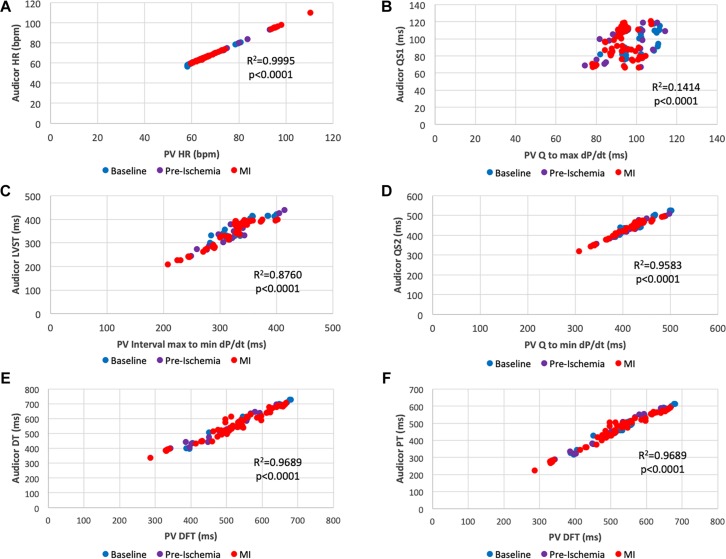
Scatter plots of overlapping interval times obtained by acoustic cardiography and pressure-volume analysis. **(A)** PV Heart Rate vs. AC Heart Rate; **(B)** Q to max dP/dt vs. QS1; **(C)** max to min dP/dt vs. S1 to S2 (LVST); **(D)** Q to min dP/dt vs. QS2; **(E)** Diastolic Filling Time vs. S2 to S1 (LVDT); **(F)** Diastolic Filling Time vs. S2 to Q (LVPT). Color key: blue (baseline), pre-ischemia (purple), red (myocardial ischemia). Each dot represents a 30-s assessment. Pressure-Volume data are reported on the *x*-axis, data obtained from acoustic cardiography are on the *y*-axis.

**FIGURE 4:**
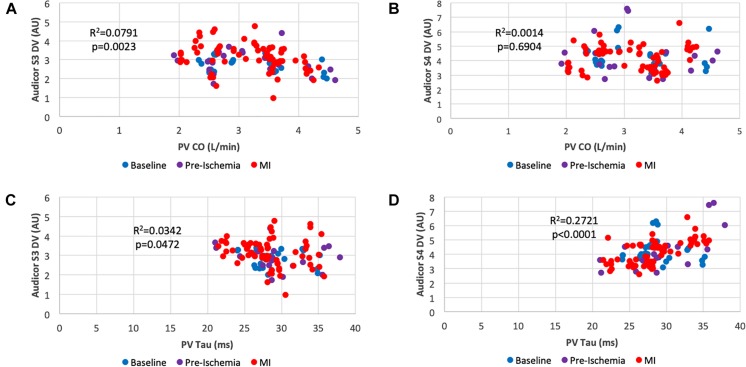
Scatter plots of systolic and diastolic performance obtained by acoustic cardiography and pressure-volume analysis. **(A)** Cardiac Output vs. S3 Display Value; **(B)** Cardiac Output vs. S4 Display Value; **(C)** Isovolumic Relaxation Costant (Tau) vs. S3 Display Value; **(D)** Isovolumic Relaxation Constant (Tau) vs. S4 Display Value. Color key: blue (baseline), pre-ischemia (purple), red (myocardial ischemia). Each dot represents a 30-s assessment. Pressure-Volume data are reported on the *x*-axis, whereas data obtained from acoustic cardiography are on the *y*-axis.

As shown in Figure 2 parameters obtained by PV loop analysis are overlapping with a number of corresponding parameters derived from AC; this figure provides a summary overview of correlated and overlapping parameters from the two techniques. In Figures 3, 4 each datapoint shown in the scatterplots represents a 30 s-averaged assessment both at baseline, pre-ischemia (time window between coronary engagement and occlusion) and each minute after coronary occlusion up to 5 min.

#### Heatmap Generation

Heatmaps were generated by comparing a constant baseline timepoint after the animal was fully instrumented and before engaging the coronary arteries with a pre-ischemic timepoint and every minute upon complete balloon inflation for 5 min as reported in Figure 1C. Fold change with respect to the aforementioned timepoint and obtained *p*-value obtained from paired *t*-test and Wilcoxon signed rank test were reported in two distinct heatmaps. Significant parameter changes at specific timepoints are indicated with an asterisk symbol in order to increase readability.

#### Area Under the Receiver Operating Characteristic Curve

In order to identify parameters that could be of diagnostic usefullness in detecting ischemic insult, we employed areas under the receiver operating characteristic (ROC) curve (AUC) for each single AC parameter during our experiment and allocated timepoints into two groups (ischemic and non-ischemic) based on the status of coronary patency. Data obtained prior to coronary engagement were considered as non-ischemic; on the other hand, data gathered after complete coronary occlusion were allocated into the ischemic group. With the intention of identifying a superior parameter in terms of predictive power we tested different models containing various combinations of the four significant single parameters. AUC was assessed for each model and the first three outperforming the single most significant one were reported. For this specific analysis, data obtained within the interval of coronary engagement and occlusion (pre-ischemia) were not employed in order to reduce a heterogenous confounding factor.

### Statistical Analysis

Continuous variables were intially tested (Shapiro-Wilk) for normal distribution. Data are presented as mean ± standard deviation or median (IQR) when appropriate. Parametric and non-parametric tests for paired comparisons between two datasets included paired *t*-test and Wilcoxon signed rank test, respectively. Areas under the receiver operating characteristic (ROC) curve (AUC) were used to quantify the predictive accuracy of AC parameters for coronary occlusion detection. Using logistic regression, the four most significant AC parameters were modeled for ischemic detection improvement.

A two-tailed *p*-value < 0.05 was considered significant. Statistical analyses were performed on GraphPad Prism 8 (GraphPad Software, San Diego, CA, United States) and STATA14 (StataCorp LP, College Station, TX, United States).

### Data and Materials Disclosure

The data that support the findings of this study are available from the authors.

## Results

During our 5 min study time-window, 3 out of 10 animals suffered from ventricular arrhythmias which required prompt defibrillation and therefore seven pigs completed the protocol. In all three cases, resuscitation maneuvers were successful and subsequent echocardiographic assessment was performed in all ten animals. Recordings were of good quality in the entire cohort. Coronary occlusion was effective in inducing significant morpho-functional alterations consistent with myocardial ischemic damage as shown in Table 1. Our porcine model was primarily characterized by systolic dysfunction [Left Ventricular Ejection Fraction (LVEF) decreased from 52.6 to 44.6%, *p* < 0.001, Left Ventricular End-Systolic Pressure (LVESP) from 73.8 to 64.9 mmHg, *p* < 0.001, Left Ventricular End-Systolic Volume (LVESV) from 45.6 mL at baseline to 56.6 mL after balloon occlusion, *p* = 0.005]. Upon experiment termination, venous blood was drawn for Troponin-T assessment in all animals and a median value of 48.5 ng/mL (41.25–211.5) was detected. Taken together these alterations prove the reliability of our working animal model and subsequently of our dataset.

**TABLE 1:** Cardiac ultrasound and PV assessment parameters prior and after coronary occlusion in 10 female pigs.

**Parameters**	**Baseline**	**Post-occlusion**	***p*-value**
	**(*n* = 10)**	**(*n* = 10)**	
**Pressure volume analysis**			
Heart rate (bpm)	62 (59.25 − 64)	64.5 (61.25 − 68.5)	0.179
Stroke volume (mL)	49.61 ± 9.00	45.62 ± 10.46	0.074
Cardiac output (L/min)	3.15 ± 0.66	3.03 ± 0.71	0.530
Ejection fraction (%)	52.62 ± 2.03	44.57 ± 4.27	<0.001
Stroke work (mmHg × mL)	2872 ± 471	2194 ± 474	0.001
Tau (ms)	28.59 ± 3.24	27.96 ± 2.60	0.389
Max dP/dt (mmHg/s)	771.19 ± 123.02	698.43 ± 134.04	0.037
Min dP/dt (mmHg/s)	−916.32 ± 170.80	−814.37 ± 169.71	0.002
End-diastolic pressure (mmHg)	13.41 ± 2.43	13.69 ± 3.16	0.725
End-systolic pressure (mmHg)	73.79 ± 9.55	64.96 ± 9.24	<0.001
End-diastolic volume (mL)	92.88 ± 16.35	100.70 ± 16.65	0.096
End-systolic volume (mL)	45.62 ± 7.78	56.62 ± 8.24	0.005
**Trans-esophageal cardiac ultrasound analysis**			
Evmax (cm/s)	49.11 ± 10.26	52.23 ± 11.92	0.419
E deceleration time (ms)	89.80 ± 27.30	73.70 ± 17.06	0.165
Avmax (cm/s)	50.53 ± 12.19	46.02 ± 13.33	0.317
E VTI (cm)	5.07 ± 1.29	4.87 ± 1.26	0.595
A VTI (cm)	2.43 ± 0.90	2.46 ± 0.95	0.934
Ea max (cm/s)	7.55 ± 2.24	7.42 ± 2.76	0.854
Aa max (cm/s)	10.81 ± 3.71	10.06 ± 2.86	0.337
Sa max (cm/s)	5.30 ± 0.91	5.46 ± 1.13	0.579
Ea VTI (cm)	0.61 ± 0.16	0.55 ± 0.23	0.368
Aa VTI (cm)	0.54 ± 0.12	0.52 ± 0.14	0.341
Sa VTI (cm)	1.18 ± 0.22	1.03 ± 0.21	0.027
E/A ratio	1.01 ± 0.22	1.20 ± 0.41	0.256
E/E’	7.07 ± 2.49	7.71 ± 2.62	0.127

*Results are reported as mean ± standard deviation or median (interquartile range) when appropriate. Paired *t*-test and Wilcoxon signed rank test were employed for normal and non-normal distributed datasets. Data obtained for post-occlusion were on average gathered after 20 min upon coronary occlusion.*

Figure 3 shows a series of scatterplots assessing the relationship between “overlapping” time parameters obtained with AC and PV. Recorded Heart Rate (HR) (Figure 3A) was similar in both detection methods (*r*^2^ = 0.9995, *p* < 0.0001). As for STIs, an overall positive correlation between parameters was found. For instance, the correlation between Electromechanical activation time (EMAT or QS1) and Q to max dP/dt (Figure 3B) was significant (*r*^2^ = 0.1414, *p* < 0.0001), nevertheless longer STIs such as left ventricular systolic time (LVST) was better correlated with its PV associated parameter (max to min dP/dt) (*r*^2^ = 0.876, *p* < 0.0001) (Figure 3C). This was valid for an additional and even longer STI EMST: QS2 strongly correlated with Q to min dP/dt (*r*^2^ = 0.9583, *p* < 0.0001) (Figure 3D).

As DTIs are concerned, AC and PV defined similar overlapping intervals: PV diastolic filling time was significantly correlated with AC perfusion time (PT or S2-Q) (*r*^2^ =0.9689, *p* < 0.0001) (Figure 3F) as well as with AC diastolic time (DT or S2-S1) (*r*^2^ = 0.9689, *p* < 0.0001) (Figure 3E).

Figure 4 shows scatterplots correlating quantifiable heart sound features (DV as explained above) with indexes of systolic and diastolic function. Cardiac output was significantly correlated with Audicor S3 DV (Figure 4A) (*r*^2^ = 0.0791, *p* = 0.0023) which was not valid for Audicor S4 DV (Figure 4B) (*r*^2^ = 0.0014, *p* = 0.6904). As for diastolic function assessment, Tau weakly correlated with Audicor S3 DV (Figure 4C) (*r*^2^ = 0.0342, *p* = 0.0472), however, a more significant correlation was found with Audicor S4 DV (Figure 4D) (*r*^2^ = 0.2721, *p* < 0.0001).

**FIGURE 5:**
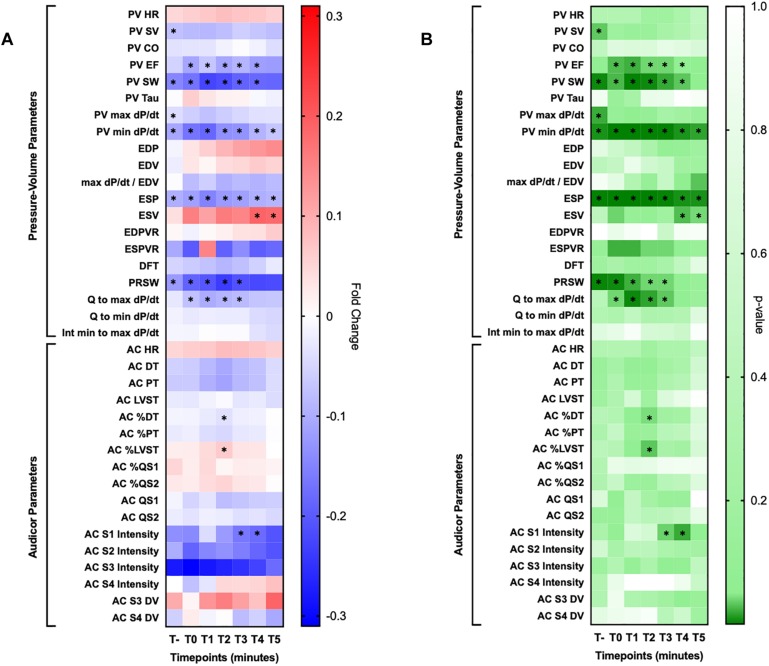
Heatmaps. **(A)** Reported fold changes obtained by comparing baseline measurement with their respective at each minute for 5 min upon coronary occlusion. **(B)** Reported two tailed *t*-test *p*-values obtained by with the same exact approach employed for the other heatmap. HR, heart rate; SV, stroke volume, CO, cardiac output, EF, ejection fraction; EDP, end diastolic pressure; EDV, end diastolic volume; ESP, end systolic pressure; ESV, end systolic volume; EDPVR, end diastolic pressure-volume relationship; ESPVR, end systolic pressure-volume relationship; DFT, diastolic filling time; PRSW, pre-recruitable stroke work; DT, diastolic time; PT, perfusion time; LVST, left ventricular systolic time; DV, display value. Significant changes are marked with an asterisk sign (^∗^).

Heatmaps provide valuable information regarding the fold change of various parameters and their respective statistical significance during the first 5 min of coronary occlusion in comparison to baseline measurements as reported in Figure 5.

Among them, PV identified these parameters as significantly changed within the 5-min observation time: left ventricular ejection fraction (LVEF) (up to −12.1%), stroke work (SW) (up to −22.7%), min dP/dt (up to −18.5%), max dP/dt/end diastolic volume (up to −9.7%), left ventricular end systolic pressure (LVESP) (up to −15.1%), left ventricular end systolic volume (LVESV) (up to +19.0%), pre-recruitable stroke work (PRSW) (up to−23.7%), Q to max dP/dt (up to −10.2%). Of note, SW, min dP/dt and ESP were significantly and constantly decreased compared to baseline reference at every timepoint during the 5 min observation window.

**FIGURE 6:**
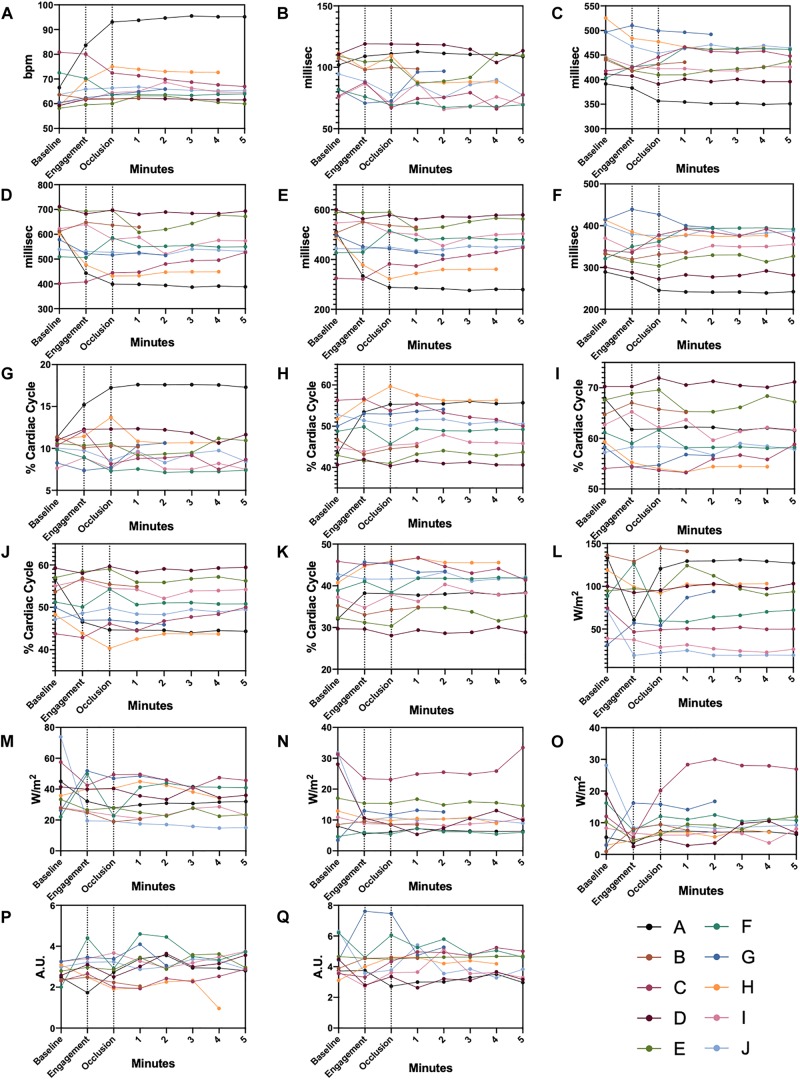
Single Datapoint Charts AC parameters shown for each animal from baseline, pre-ischemia, and ischemia phases. Dashed lines delimit the three different experimental steps. **(A)** heart rate, **(B)** QS1, **(C)** QS2, **(D)** Diastolic time, **(E)** Perfusion time, **(F)** Left ventricular systolic time, **(G)** QS1 shown in percentage of a complete cardiac cycle, **(H)** QS2 shown in percentage of a complete cardiac cycle, **(I)** Diastolic time shown in percentage of a complete cardiac cycle, **(J)** Perfusion time shown in percentage of a complete cardiac cycle, **(K)** Left ventricular systolic time shown in percentage of a complete cardiac cycle, **(L)** S1 intensity, **(M)** S2 intensity, **(N)** S3 intensity, **(O)** S4 intensity, **(P)** S3 display value, **(Q)** S4 display value. Color-coded legends are provided for single animal identification.

On the other hand, AC identified the following parameters as significantly changed in comparison to baseline: diastolic time percentage (DT%) (up to −3.7%), left ventricular systolic time percentage (up to + 6.3%) and first heart sound intensity (up to −20.8%). Single dataset trends over our observation window for AC parameters are presented for clarity in Figure 6.

**FIGURE 7:**
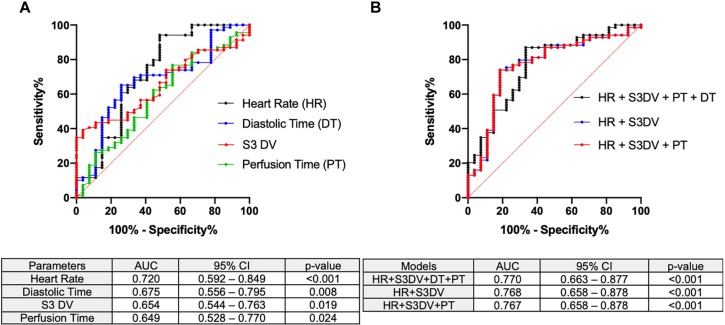
Area under the receiver operating characteristic curves AC parameters were tested for ischemic detection. AUC, 95% confidence interval and *p*-values are included. **(A)** The significant single AC parameters (HR, PT, DT, S3DV) are here reported. **(B)** Combinatory models derived from logistic regression outperforming single parameters are reported.

As anticipated, Figure 7A shows the four significant AC parameters in terms of predictive power in detecting an ischemic condition: Heart Rate (AUC 0.720, CI95% 0.592–0.849, *p* < 0.001), Diastolic Time (AUC 0.675, CI95% 0.556–0.795, *p* = 0.008), S3 DV (AUC 0.654, CI95% 0.544–0.763, *p* = 0.019), Perfusion Time (AUC 0.649, CI95% 0.528–0.770, *p* = 0.024). With the intention of identifying a more powerful joint-index that could outperform any of the above mentioned single parameters we employed models upon linear regression with different combinations of the four AC parameters shown in Figure 7A. Figure 7B shows the three most significant joint-indexes: HR + DT + PT + S3DV (AUC 0.770, CI95% 0.663–0.877, *p* < 0.001), HR + S3DV (AUC 0.768, CI95% 0.658–0.878, *p* < 0.001), HR + PT + S3DV (AUC 0.767, CI95% 0.658–0.878, *p* < 0.001).

## Discussion

### Reintroducing an Old Technique

Cardiac auscultation has known a crescendo-decrescendo use trend in the medical community. Despite acknowledging the usefulness of information obtained by auscultatory examination, other diagnostic methods are usually preferred. Nevertheless the digitalization, in addition to the ECG coupling has propelled new interest in acoustics in light of the minimal invasiveness of such technique. Being already proven as an effective diagnostic and prognostic method, we envisioned a diagnostic role of AC in the setting of myocardial ischemia, a well-known condition characterized by mechanical dysfunction that could be detectable in the first minutes of coronary occlusion. In the past, different investigations have led to divergent results in light of heterogeneous infarct sizes, adrenergic tone, time of infarct onset and adopted methods of detection (Hamosh et al., 1972; Weissler, 1987).

In order to overcome a number of these biases, we employed a porcine model of AMI by occluding the coronary artery at similar levels of the coronary tree thus reducing confounding factors in a stable and rapidly evolving condition by dissecting the role of acoustic changes and derivative parameters in AMI.

Table 1 offers a rapid overview of myocardial dysfunction observed in our porcine model. These significant changes are observed on average 20 min after coronary occlusion and they are not expected to change to this magnitude in the very first minutes during ischemia. However, our multimodal realtime recording allows the detection of several parameters that can rapidly change upon ischemia and might represent candidates of damage sensing.

### Systolic Time Intervals

Contractile dysfunction can be detected by abnormalities in force generation, wall shortening, ejected blood volume and temporal relationships of contraction phases (Boudoulas, 1990).

As shown in the heatmaps in Figure 5, indexes of dysfunction were already detectable in the first minute after occlusion and permanently present with invasive techniques (SW, LVESP, min dP/dt, PRSW). In regard to STIs, their correlations were significant between the techniques employed in our study, particularly for longer duration intervals (Figures 3C,D).

As extensively described by Lewis, STIs bear limited diagnostic significance in AMI compared to chronic conditions particularly if no pre-existing comorbidities are present, as in the case of our study (Lewis et al., 1977). In the setting of myocardial infarction, the shortening of QS1 is paradoxically coupled with an increase of QS percentage (QS1%). Despite being non-significant, both trends are not mutually exclusive, in fact they are in accordance with the rationales to which the absolute shortening (QS1) is driven by an adrenergic burst and the relative prolongation (QS1%) is due to diminished rate of increase in LV pressure in isolvolumic contraction (Boudoulas, 1990). Unfortunately, max dP/dt abruptly decreases in the first seconds of ischemia (10% relative reduction) but it is not coupled with a relevant QS1% magnitude increase (4% relative increase) supporting the notion that STIs, especially EMAT (QS1), should be solely employed for prognosis in convalescence phase (Diamant and Killip, 1970) rather than diagnosis in the acute setting of myocardial infarction (Hamosh et al., 1972; Hodges et al., 1972). On the other hand, PV assessment rapidly identified significant shortening of the compared STI (Q to max dP/dt) proving a superior sensitivity over AC for these specific intervals (Figure 3B) The main difference observed in this suboptimal comparison relies on the fact that the interval Q to max dP/dt, also known as Pre-Ejection Period (PEP), is a much longer interval, which encompasses EMAT, and it is markedly affected by systolic dysfunction, whilst EMAT is a shorter interval that is majorly prolonged in case of conduction defect.

### Diastolic Time Intervals

With regard to DTIs, our study provides evidence of significant correlations between PV and AC analysis in identifying overlapping intervals. Of note is the significant reduction observed in diastolic time (expressed as percentage within the entire cardiac cycle) upon coronary occlusion even with a non-invasive method, therefore configuring itself as a possible criterion for ischemic damage. Despite being significant for a single timepoint, statistical significance was reached with a relatively small sample as our cohort and more importantly this finding is not unexpected. In fact, clinical investigations conducted by Ferro et al. (1991, 1995) have proven the relevance of diastolic time in assessing degree of coronary occlusion and stress-induced ischemic threshold. In support of such findings, AUC of ROC identified both diastolic and perfusion times (reported as unit of time) as possible parameters with clinical signficance in detecting ischemic acute event.

Whether reported as unit of time or as a ratio, diastolic duration, appears as pivotal indicator of ischemic damage independently of the primary limiting factor: coronary lumen patency or vasodilator capacity in coronary artery disease or syndrome X patients, respectively.

### Heart Sounds

The prominent feature of AC is certainly the quantification of heart sound properties, particularly diastolic ones, which are of difficult interpretation even for experienced clinicians (Marcus et al., 2006; Vukanovic-Criley et al., 2006).

The S3 and S4 heart sounds are diastolic filling cardiac sounds generated for a series of pathological causes. They are validated as relatively specific and non-invasive indicators of heart failure (Shah et al., 1968) and found to appear during acute ischemia (Harris et al., 2006). The former is caused by a limited longitudinal expansion of the ventricular wall during early diastolic filling yielding to a rapid deceleration of ejected blood (Ozawa et al., 1983a, b) and it correlates with parameters of impaired diastolic function in particular left ventricular filling pressures (Kono et al., 1993; Tribouilloy et al., 2001; Horwich et al., 2003). Not surprisingly, in our study, S3 DV rapidly increases (+ 19% at minute 5) along with augmented filling pressures (LVEDP + 14% at minute 5) consistent with the body of literature produced on this subject in the last decades. Out of a series of indexes of systolic function S3 DV better and inversely correlated with Cardiac Output (Figure 4A). In addition, S3 DV itself was proven as a statistically significant parameter (*p* = 0.019, Figure 7) for ischemic damage detection.

On the other hand S4, is a better indicator of diastolic stiffness and our dataset confirms such axiom as S4 DV positively and significantly correlated with the isovolumic relaxation constant (Tau) (Figure 4D). These findings are in line with the current knowledge in physiology proving a degree of specificity of these two heart sounds in detecting two contemporary yet distinct defects occurring during AMI.

### Study Limitations

The current study has a number of contemplated limitations: here we presented a pre-clinical study with a relatively small cohort composed of healthy animals under general anesthesia. Despite the abovementioned limitations, our experience sheds light on putative immediate mechanical alterations that would be otherwise unfeasible to replicate in a real-world clinical scenario and it might represent a reference point for future studies involving AC with more complex and heterogenous conditions.

## Conclusion

In conclusion, the present study provides novel and preliminary evidence of the usefulness, reliability and responsiveness of AC in detecting mechanical defects (particularly S3 heart sound and DTIs) appearing in the very first minutes during coronary occlusion. This responsive detection might have a possible application in peculiar patient subpopulations who would benefit from a chronic, non-invasive monitoring approach. Lastly, the digitalization of this hybrid technique will allow the quantification of acoustic parameters which could be of great diagnostic importance (Schwartz and Little, 1961) in an unbiased manner and, more importantly, independently of operator’s expertise (Mangione and Nieman, 1997) and propel new interest toward this non-invasive method.

## Ethics Statement

The animal housing and experimental protocols were approved by the Cantonal Veterinary Department, Zurich, Switzerland, under license no. ZH 219/2016, and were in accordance with Swiss Animal Protection Law. Housing and experimental procedures also conformed to the European Directive 2010/63/EU of the European Parliament and of the Council on the Protection of Animals used for scientific purposes and to the Guide for the Care and Use of Laboratory Animals (Institute of Laboratory Animal Resources, National Research Council, National Academy of Sciences, 2011).

## Author Contributions

NC, PB, AG, ER, PE, MZ, and FM conceived and designed the study. MaL, MS, SK, PA, PB, and MZ analyzed the data. MaL and PA performed the statistical analysis. MaL wrote the first draft of the manuscript. NC, MiL, PA, and MZ wrote the sections of the manuscript. All authors contributed to the manuscript revision, and read and approved the submitted version.

## Conflict of Interest Statement

PA and PB work for VisCardia, Inc., United States. The remaining authors declare that the research was conducted in the absence of any commercial or financial relationships that could be construed as a potential conflict of interest.
